# Safety of total body irradiation using intensity-modulated radiation therapy by helical tomotherapy in allogeneic hematopoietic stem cell transplantation: a prospective pilot study

**DOI:** 10.1093/jrr/rraa078

**Published:** 2020-09-05

**Authors:** Tatsuya Konishi, Hiroaki Ogawa, Yuho Najima, Shinpei Hashimoto, Atsushi Wada, Hiroto Adachi, Ryosuke Konuma, Yuya Kishida, Akihito Nagata, Yuta Yamada, Satoshi Kaito, Junichi Mukae, Atsushi Marumo, Yuma Noguchi, Takashi Toya, Aiko Igarashi, Takeshi Kobayashi, Kazuteru Ohashi, Noriko Doki, Katsuyuki Karasawa

**Affiliations:** Hematology Division, Tokyo Metropolitan Cancer and Infectious Diseases Center, Komagome Hospital; Division of Radiation Oncology, Department of Radiology, Tokyo Metropolitan Cancer and Infectious Diseases Center, Komagome Hospital; Hematology Division, Tokyo Metropolitan Cancer and Infectious Diseases Center, Komagome Hospital; Division of Radiation Oncology, Department of Radiology, Tokyo Metropolitan Cancer and Infectious Diseases Center, Komagome Hospital; Hematology Division, Tokyo Metropolitan Cancer and Infectious Diseases Center, Komagome Hospital; Hematology Division, Tokyo Metropolitan Cancer and Infectious Diseases Center, Komagome Hospital; Hematology Division, Tokyo Metropolitan Cancer and Infectious Diseases Center, Komagome Hospital; Hematology Division, Tokyo Metropolitan Cancer and Infectious Diseases Center, Komagome Hospital; Hematology Division, Tokyo Metropolitan Cancer and Infectious Diseases Center, Komagome Hospital; Hematology Division, Tokyo Metropolitan Cancer and Infectious Diseases Center, Komagome Hospital; Hematology Division, Tokyo Metropolitan Cancer and Infectious Diseases Center, Komagome Hospital; Hematology Division, Tokyo Metropolitan Cancer and Infectious Diseases Center, Komagome Hospital; Hematology Division, Tokyo Metropolitan Cancer and Infectious Diseases Center, Komagome Hospital; Hematology Division, Tokyo Metropolitan Cancer and Infectious Diseases Center, Komagome Hospital; Hematology Division, Tokyo Metropolitan Cancer and Infectious Diseases Center, Komagome Hospital; Hematology Division, Tokyo Metropolitan Cancer and Infectious Diseases Center, Komagome Hospital; Hematology Division, Tokyo Metropolitan Cancer and Infectious Diseases Center, Komagome Hospital; Hematology Division, Tokyo Metropolitan Cancer and Infectious Diseases Center, Komagome Hospital; Hematology Division, Tokyo Metropolitan Cancer and Infectious Diseases Center, Komagome Hospital; Division of Radiation Oncology, Department of Radiology, Tokyo Metropolitan Cancer and Infectious Diseases Center, Komagome Hospital

**Keywords:** total body irradiation, helical tomotherapy, intensity-modulated radiation therapy, allogeneic stem cell transplantation

## Abstract

Total body irradiation using intensity-modulated radiation therapy total body irradiation (IMRT-TBI) by helical tomotherapy in allogeneic hematopoietic stem cell transplantation (allo-HSCT) allows for precise evaluation and adjustment of radiation dosage. We conducted a single-center pilot study to evaluate the safety of IMRT-TBI for allo-HSCT recipients. Patients with hematological malignancies in remission who were scheduled for allo-HSCT with TBI-based myeloablative conditioning were eligible. The primary endpoint was the incidence of adverse events (AEs). Secondary endpoints were engraftment rate, overall survival, relapse rate, non-relapse mortality, and the incidence of acute and chronic graft-versus-host disease (aGVHD and cGVHD, respectively). Between July 2018 and November 2018, ten patients were recruited with a median observation duration of 571 days after allo-HSCT (range, 496–614). D_80%_ for planning target volume (PTV) in all patients was 12.01 Gy. Average D_80%_ values for lungs, kidneys and lenses (right/left) were 7.50, 9.03 and 4.41/4.03 Gy, respectively. Any early AEs (within 100 days of allo-HSCT) were reported in all patients. Eight patients experienced oral mucositis and gastrointestinal symptoms. One patient experienced Bearman criteria grade 3 regimen-related toxicity (kidney and liver). All cases achieved neutrophil engraftment. There was no grade III–IV aGVHD or late AE. One patient died of sinusoidal obstruction syndrome 67 days after allo-HSCT. The remaining nine patients were alive and disease-free at final follow-up. Thus, IMRT-TBI was well tolerated in terms of early AEs in adult patients who underwent allo-HSCT; this warrants further study with longer observation times to monitor late AEs and efficacy.

## INTRODUCTION

Allogeneic hematopoietic stem cell transplantation (allo-HSCT) is a potentially curative treatment for hematological diseases, and total body irradiation (TBI) has been commonly used as part of the conditioning regimen during the last three decades [1]. TBI for allo-HSCT has powerful immunosuppressive effects and cytotoxicity, notably towards malignant hematopoietic cells that evade chemotherapy [2, 3]. However, TBI may cause various adverse events (AEs) such as radiation sickness, rash and mucositis in the early stages, and radiation pneumonitis, renal dysfunction, cataracts, infertility, endocrine disorders and secondary malignancies in the late stages [4–7]. Late-stage AEs are irreversible and sometimes fatal, so the total dose of standard TBI for allo-HSCT is 12 Gy in six fractions over 3 days [8, 9].

Intensity-modulated radiation therapy (IMRT) can achieve a radiation dose distribution with high conformity to the target while avoiding risk to adjacent organs [10]. Compared to conventional TBI methods (e.g. moving couch method or long source-to-skin distance method), IMRT allows for precise evaluation and adjustment of the radiation doseage. Moreover, IMRT, together with image-guided radiotherapy (IGRT), delivers more accurate treatment. Helical tomotherapy (HT) is a system of radiation therapy delivery designed to perform IMRT with IGRT. TBI with IMRT (IMRT-TBI) using HT is widely adaptable, making these flexible treatments available to a variety of patients.

Recently, some reports have indicated that conditioning patients with hematological malignancies via IMRT-TBI (total 12 Gy) resulted in no severe acute toxicity or increased cumulative incidence of relapse (CIR) [11, 12]. However, the safety and efficacy of IMRT-TBI remain unclear because of the limited numbers of studied cases. Although there is a published simulation study investigating the adequate target volume for IMRT-TBI [13], there are no reports that explicitly investigate the safety of IMRT-TBI in allo-HSCT recipients in Japan. Therefore, we evaluated the safety of an IMRT-TBI-based myeloablative conditioning regimen for allo-HSCT recipients in our hospital.

## MATERIALS AND METHODS

### Study design

The present study was a single-center pilot study for patients with hematological malignancies in complete remission before allo-HSCT. The primary endpoint was the incidence of AEs according to Bearman’s criteria [14] and the National Cancer Institute Common Terminology Criteria for Adverse Events (CTCAE) version 4.0. Secondary endpoints were engraftment rate, overall survival (OS), disease-free survival (DFS), CIR, non-relapse mortality (NRM) and the incidence of acute or chronic graft-versus-host disease (aGVHD or cGVHD, respectively).

The ethical committee of the Tokyo Metropolitan Cancer and Infectious Diseases Center, Komagome Hospital approved this study (reference number 2138). This study is registered in the University Hospital Medical Information Network Clinical Trials Registry (UMIN-CTR: UMIN 000033248). All subjects provided written, informed consent.

### Patients

Patients were recruited between July 2018 and November 2018 and followed up until 31 March 2020. We included patients at our hospital aged <60 years who were scheduled to undergo 12 Gy TBI as a myeloablative conditioning regimen before allo-HSCT. Eligible patients were required to have an Eastern Cooperative Oncology Group performance status of <3, a cardiac ejection fraction of ≥50%, percent vital capacity and forced expiratory volume in 1 s of ≥70%, serum bilirubin of ≤2 mg/dL, alanine aminotransferase and aspartate aminotransferase of up to 5-fold higher than the upper limits of normal, and a calculated creatinine clearance of ≥30 mL/min/m^2^. We excluded patients who had an extramedullary disease, a history of allo-HSCT or autologous transplantation, another malignancy, uncontrolled diabetes mellitus or who were pregnant.

### Procedures and definition

The TomoTherapy Radixact™ (Accuray, Inc., Madison, WI, USA), a new generation HT platform, was used for TBI in all subjects. The planning computed tomography (CT) was performed ~2 weeks before TBI. Patients were immobilized using a full-body evacuated cushion (CIVCO Medical Solutions, Coralville, IA, USA) and a thermoplastic mask (CIVCO Medical Solutions) over the head and neck in a stable, supine position. Treatment planning CT images were obtained with a slice thickness of 5 mm. In the present study, the lungs, kidneys and lenses were selected as the risk organs as these have been implicated for shielding in conventional TBI. The clinical target volume (CTV) was defined as the whole body excluding the risk organs. The planning target volume (PTV) was defined as equivalent to the CTV. The prescribed dose for TBI was 12 Gy in 6 fractions twice daily for three consecutive days, with an interval between fractions ≥6 h. For the PTV, the minimum doses received by 80% (D_80%_) and maximum doses (D_max_) received were set 98–105 and 115%, respectively, of the prescription doses. Dose restrictions for the risk organs were determined as follows: average doses <8 Gy and a minimum dose received by 2% (D_2%_) <12 Gy in the lungs; average doses <10 Gy and a D_2%_ <12 Gy in the kidneys; average doses <6 Gy and a D_max_ <10 Gy in the lenses. Due to the maximum movement range of the Radixact™ bed, the radiation field was divided into two parts: the head side and foot side. The gap between the two fields was adjusted with reference to dose distributions, to minimize the volume exceeding 110% of the prescription dose. Dose verification of the treatment plan was performed according to the physical technology guidelines of IMRT published by the Japan Society of Radiation Oncology [15]. TBI was deliverd with image-guided radiation therapy, and whole-body mega-voltage CT was used to localize the patients.

The standard conditioning regimen consisted of cyclophosphamide (CY, 60 mg/kg/day) for 2 days and IMRT-TBI (12 Gy) for 3 days. As suggested by existing literature, additional cytarabine or etoposide administration was at the discretion of the attending physician [16–19]. Standard GVHD prophylaxis consisted of a calcineurin inhibitor (cyclosporine or tacrolimus) with short-term methotrexate or mycophenolate mofetil. Anti-thymocyte globulin as GVHD prevention was administered at the discretion of the attending physician [20]. We defined neutrophil engraftment as the first of three consecutive days when absolute neutrophil count was ≥0.5 × 10^9^/L. We defined platelet engraftment as the first of seven consecutive days when platelet count was ≥50 × 10^9^/L without transfusion support. Acute and chronic GVHD were diagnosed and graded using previously established criteria [21, 22]. Causes of death were classified based on a previous report [23].

## RESULTS

### Patient characteristics and radiation dose measurement

Patient characteristics are presented in Table 1. Ten patients were recruited and underwent allo-HSCT between July 2018 and November 2018. The median age was 45.5 years (range, 20–53), with six males and four females. The median follow-up period for survivors was 571 days (range, 496–614). Underlying diseases were acute lymphoblastic leukemia (*n* = 4), acute myeloid leukemia (*n* = 2), mixed phenotype acute leukemia (*n* = 2) and chronic myeloid leukemia (*n* = 2). All patients had received some chemotherapy and were in complete remission or chronic phase before allo-HSCT.

**Table 1:** Patient characteristics^a^

Case	Age/sex	Disease	Disease status before HSCT	Donor source	Stem cell source	HLA disparity	PS before HSCT	HCT-CI	Conditioning regimen	GVHD prophylaxis
1	29/M	ALL	1st CR	Unrelated	BM	7/8	0	0	CY + TBI 12 Gy	FK + sMTX
2	46/M	MPAL	2nd CR	Unrelated	CB	6/8	2	0	VP-16 + CY + TBI 12 Gy	FK + sMTX
3	49/F	ALL	1st CR	Unrelated	BM	8/8	0	2	CY + TBI 12 Gy	FK + sMTX
4	20/M	ALL	1st CR	Unrelated	BM	8/8	0	2	CY + TBI 12 Gy	FK + sMTX
5	49/F	MPAL	1st CR	Unrelated	BM	8/8	1	0	CY + TBI 12 Gy	FK + sMTX
6	23/F	AML	1st CR	Unrelated	PB	8/8	0	0	CA + CY + TBI 12 Gy	FK + sMTX
7	39/M	AML	1st CR	Related	PB	8/8	0	1	CA + CY + TBI 12 Gy	CsA + sMTX
8	45/F	CML	2nd CP	Unrelated	BM	7/8	1	0	CY + TBI 12 Gy	FK + sMTX + ATG
9	53/M	CML	2nd CP	Unrelated	BM	8/8	0	3	CY + TBI 12 Gy	FK + sMTX
10	50/M	ALL	1st CR	Unrelated	BM	8/8	0	3	CY + TBI 12 Gy	FK + sMTX

^a^ALL = acute lymphoblastic leukemia, AML = acute myeloid leukemia, ATG = anti-thymocyte globulin, BM = bone marrow, CA = cytarabine, CB = cord blood, CML = chronic myeloid leukemia, CP = chronic phase, CR = complete remission, CsA = cyclosporine, F = female, FK = tacrolims, HCT-CI = hematopoietic cell transplantation specific comorbidity index, HLA = human leukocyte antigen, M = male, MPAL = mixed phenotype acute leukemia, PB = peripheral blood, PS = performance status, sMTX = short term methotrexate, VP-16 = etoposide.

In our conditioning regimen, the chemotherapy paired with IMRT-TBI was either CY (*n* = 7), cytarabine + CY (*n* = 2) or etoposide + CY (*n* = 1). Two patients (cases 2 and 7) underwent TBI before chemotherapy, and the remaining eight patients were irradiated for 3 days before allo-HSCT (day −3 to −1 or day −2 to 0). Seven patients had human leukocyte antigen (HLA)-matched transplants and one patient received allo-HSCT from a related donor.

**Fig. 1.**
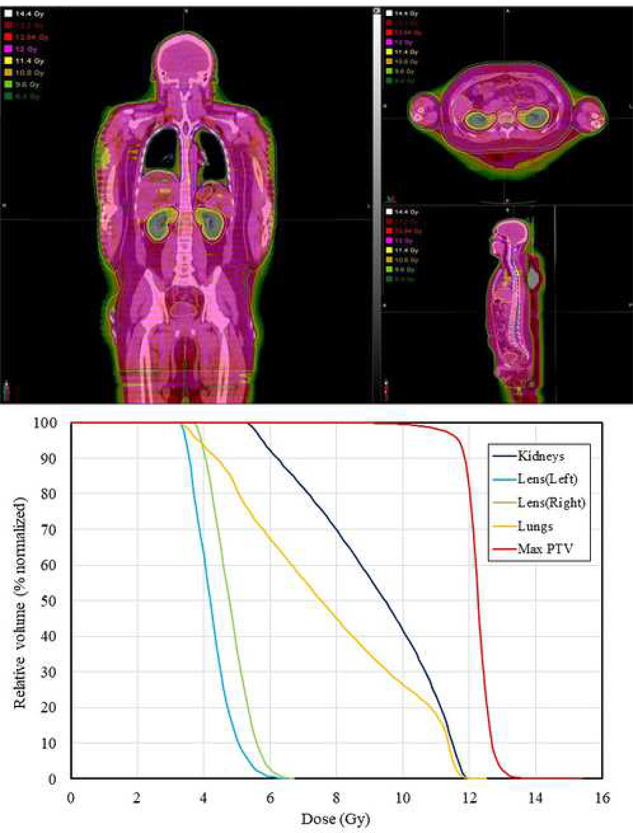
Dose distribution and dose–volume curves in a representative case.

Radiation information and radiation dose parameters are described in Tables 2 and 3, respectively. A pitch of 0.397 was selected in most patients due to shorter beam-on time, however a pitch of 0.287 was thought to be optimal for some patients with small body size to provide better dose homogeneity [24, 25]. The median D_80%_ of PTV in all patients was 12.01 Gy (range, 11.97–12.05). The dose distribution and the dose volume of an exemplary case are presented in Fig. 1. The average D_80%_ values of for lungs, kidneys and lenses (right/left) were 7.50, 9.03 and 4.41/4.03 Gy, respectively. The treatment plans for all patients satisfied dose constraints.

**Table 2:** Radiation therapy information

Case	Head side treatment					Foot side treatment				
	Beam on time (s)	Pitch	MF^a^	Jaw (cm)	Image acquistion time (s)	Beam on time (s)	Pitch	MF	Jaw (cm)	Image acquistion time (s)
1	1560	0.287	2	5	480.5	780.9	0.287	2	5	375.5
2	1132.5	0.397	2.1	5	501.5	535.6	0.397	2	5	369.5
3	1121.6	0.397	2.2	5	462.5	458	0.397	2	5	279.5
4	1083.6	0.397	2.2	5	459.5	705.1	0.397	2	5	303.5
5	988.7	0.397	2.1	5	441.5	428.8	0.397	2	5	273.5
6	1071.9	0.397	2.2	5	465.5	522.4	0.397	2	5	282.5
7	1127.3	0.397	2.2	5	498.5	570.8	0.397	2	5	291.5
8	987.1	0.397	2.1	5	429.5	577.9	0.397	2	5	270.5
9	1008.6	0.397	2.1	5	435.5	595.6	0.397	2	5	282.5
10	1057.6	0.397	2.15	5	471.5	639.5	0.397	2	5	288.5

^a^MF = moduration factor.

**Table 3:** Dose measurement results

Case	PTV		Lungs		Kidneys		Lens (Right)		Lens (Left)	
	D80%	Dmax	Mean	Dmax	Mean	Dmax	D80%	Dmax	D80%	Dmax
1	11.97	14.77	7.35	12.09	8.95	12.17	4.97	6.29	4.45	6.10
2	12.05	14.94	7.51	12.54	8.99	12.36	5.24	7.40	4.87	7.50
3	12.01	14.95	7.54	12.52	9.13	12.36	4.31	6.14	3.76	5.52
4	12.01	15.42	7.72	12.42	9.16	12.34	4.81	6.64	4.26	6.43
5	12.03	14.47	7.31	12.55	9.31	12.13	3.22	5.45	3.23	4.93
6	12.01	14.11	7.49	12.61	9.1	12.35	5.86	8.02	5.78	7.07
7	12.01	14.41	7.60	12.49	8.5	12.05	4.32	6.33	3.70	6.20
8	11.97	14.74	7.43	12.58	8.98	12.27	3.68	5.33	4.01	5.44
9	12.01	14.45	7.47	12.56	9.01	11.94	3.42	5.51	2.63	4.25
10	12.02	14.66	7.53	12.59	9.14	12.34	4.22	6.62	3.65	6.08
Mean	12.01	14.69	7.50	12.50	9.03	12.23	4.41	6.37	4.03	5.95
Median	12.01	14.70	7.50	12.55	9.06	12.31	4.32	6.31	3.89	6.09

### Clinical outcomes

Clinical outcomes are shown in Table 4. The median times to neutrophil and platelet engraftment were 18.5 and 39 days, respectively. Although all patients achieved neutrophil engraftment, one patient (case 7) died of sinusoidal obstruction syndrome (SOS) before platelet recovery. At final follow-up, nine of ten patients were alive and disease-free. All grade and grade II aGVHD were observed in four and three patients, respectively. Although one patient (case 1) developed mild cGVHD, there were no cases of grade III–IV aGVHD.

**Table 4:** Clinical outcomes^a^

Case	Time to engraftment, neutrophil/platelet (days)	Time from HSCT to discharge (days)	Regimen-related toxity according to CTCAE (grade)	Early complications (within 100 days after allo-HSCT)	Late complications (last follow-up date from day100)	aGVHD, max grade (organ, stage)	cGVHD, organ (severity)	Relapse	Outcome	OS after HSCT (days)	Cause of death
1	18/39	49	Oral mucositis (3), diarrhea (3), anorexia (3), nausea (3)	-	-	II (Skin 3)	Mouth (mild)	No	Alive	614	-
2	32/42	104	Oral mucositis (2), diarrhea (2), anorexia (3), nausea (3)	SOS, Psoas hematoma, CKD	-	II (Skin 1; liver 1)	-	No	Alive	600	-
3	21/28	61	Oral mucositis (3), diarrhea (3)	Hemorrhagic cystitis	-	I (Skin 1)	-	No	Alive	599	-
4	16/30	44	Oral mucositis (3), diarrhea (3), anorexia (3), nausea (3)	-	-	I (Skin 1)	-	No	Alive	580	-
5	19/19	48	Diarrhea (1), anorexia (3)	-	-	I (Skin 1)	-	No	Alive	571	-
6	18/39	46	Diarrhea (2), nausea (3), malaise (2)	CRBSI, CDI	-	-	-	No	Alive	564	-
7	17/NE	NE	Oral mucositis (3), nausea (3)	BSI, Hemorrhagic cystitis, HPS, SOS, AKI	-	-	-	No	Dead	67	SOS
8	25/223	61	-	-	-	-	-	No	Alive	509	-
9	16/21	36	Diarrhea (1)	-	-	I (Skin 2)	-	No	Alive	503	-
10	33/134	98	Oral mucositis (3), dermatitis (3)	AKI	-	II (Skin 3)	-	No	Alive	496	-

^a^AKI = acute kidney injury, BSI = blood stream infection, CDI = *Clostridium difficile* infection, CKD = chronic kidney disease, CRBSI = catheter-related blood stream infection, HPS = hemophagocytic syndrome, NE = not evaluable.

### Adverse events

All patients reported any early AEs, which were defined as those occurring within 100 days of allo-HSCT. Conditioning-related AEs as defined by the Bearman criteria are presented in Table 5. The most common toxicity was oral mucositis, which was observed in seven patients, but no patient developed a higher grade than 2. Grade 3 toxicity based on Bearman criteria was observed in one patient (kidney and liver, case 7). Eight patients reported oral mucositis and gastrointestinal toxicities higher than CTCAE grade 3 (Table 3). One patient (case 10) suffered CTCAE grade 3 dermatitis on both legs on day four. We applied topical steroid therapy, which gradually healed the patient but caused pigmentation after neutrophil engraftment. SOS (cases 2 and 7) and hemorrhagic cystitis (cases 3 and 7) were each observed in two cases as early complications. Other than the fatal development of SOS in case 7, treatments were successful and resolved without sequelae. There were no late AEs or complications during the observation period.

**Table 5:** Adverse events according to Bearman criteria

Toxicity, *n*	Any grade	Grade 3	Grade 4
Heart	1	0	0
Bladder	2	0	0
Kidneys	4	1	0
Lungs	0	0	0
Liver	2	1	0
Central nervous system	1	0	0
Mucosa	7	0	0
Gut	3	0	0

### Clinical course of fatal SOS

Case 7 suffered from an early-phase bloodstream infection (day 5), hemorrhagic cystitis (Bearman criteria grade 2; day 17) and hemophagocytic syndrome (day 23) after allo-HSCT. On day 23, weight gain and ascites presented after neutrophil engraftment. Afterwards, serum transaminase and bilirubin levels increased with hepatomegaly, and the patient was diagnosed with SOS (grade 4) on day 27. We treated the patient with defibrotide and proper fluid balance management, but both were discontinued due to bleeding tendencies. The patient also developed acute renal failure and septic shock during the SOS treatment course, ultimately causing death on day 67.

## DISCUSSION

To our knowledge, this is the first prospective study to evaluate the safety and clinical outcomes of IMRT-TBI-based conditioning in Japanese patients receiving allo-HSCT. All patients ultimately received radiation therapy without problems. One patient developed fatal SOS, but the remaining nine survived for longer than 1 year without severe TBI-related complications. IMRT-TBI allowed for accurate dose evaluation and adjustment compared with conventional TBI methods, and it was well tolerated in the early phase in allo-HSCT patients. The treatment plan for all cases satisfied safe dose constraints, including for target volume and risk organs. Moreover, all radiation therapy was completed without any technical problems. These findings indicate that IMRT-TBI using HT is a feasible option for Japanese patients.

Two previous studies have evaluated the clinical feasibility of IMRT-TBI [11, 12]. Gruen *et al*. reported no grade 3–4 AEs in ten juvenile patients with acute leukemia. Two of ten patients in their study died of bacterial sepsis and GVHD within 3 months of allo-HSCT, and the follow-up period for survivors was ≤15 months [11]. Penagaricano *et al*. reported four adult patients with acute myeloid leukemia that received IMRT-TBI-based conditioning. Although TBI-related toxicity assessment identified only grade 1 radiation dermatitis and headache, two of four patients died of GVHD within 6 months of allo-HSCT [12]. Regarding complications, TBI dose rate has been suspected to cause lung and renal toxicities in conventional TBI [26–31]. The maximum dose rate for HT TBI (1000 cGy/min) is much higher than conventional TBI. Although several reports have described the clinical outcomes of IMRT-TBI and total marrow irradiation using HT or other radiotherapy platform [12, 32, 33], so far, no study has reported an increase in lung and renal complications. In line with the earlier reports, no unexpected severe AEs were observed in the present study, though the frequency of late AEs remains unknown.

In this study, two cases developed SOS, a severe hepatic complication after HSCT [5]. In one patient (case 2) with mixed phenotype acute leukemia in second remission, allo-HSCT was performed after two courses of inotuzumab ozogamicin (InO) administered subsequent to disease relapse following standard combination chemotherapy. Conventional TBI-based myeloablative conditioning regimens and previous InO treatments are significant risk factors of SOS (odds ratio 2.8 and 22) [34, 35]. Considering these two factors, case 2 was especially at risk of SOS. Another patient that developed SOS (case 7) received cytarabine + CY + TBI as an intensified conditioning regimen. In this study, two of the three patients who received intensified conditioning developed SOS. However, previous reports have shown that the addition of cytarabine or etoposide does not increase complication risk [16–19]. In fact, regimen-related toxicities other than SOS for these three patients were not serious and were comparable to the toxicities of the other seven patients. Although the number of patients in this study was limited, physicians should assess underlying disease or previous treatments as risk factors before indicating intensified conditioning beyond standard CY + TBI.

Some of our patients experienced CTCAE grade 3 toxicities (e.g. oral mucositis, diarrhea, anorexia and nausea; Table 3). Other than the SOS described above, we observed no grade 4 toxicities based on CTCAE or Bearman criteria, suggesting that the incidence of life-threatening regimen-related toxicities from an IMRT-TBI-based regimen is comparable to that of a conventional TBI-based regimen [15–18]. Furthermore, no late complications have been confirmed to date.

This study has several limitations. First, it was a single-institution study and consisted of a small number of cases. Second, there were some variations in the conditioning regimen; therefore, it is difficult to determine whether TBI caused any particular AE. Notably, there is yet to be an association between TBI and SOS. Third, the follow-up period (~1.5 years) is relatively short, and we only evaluated early complications. We hope that this prospective pilot study in Japan will lead to practical clinical use with extended observation periods.

In summary, IMRT-TBI was well tolerated in terms of early AEs in adult patients who underwent allo-HSCT. Based on the results of this pilot study, our hospital is currently implementing 12 Gy IMRT-TBI as a conditioning regimen instead of the conventional 12 Gy TBI. Extended observation periods focusing on late AEs are warranted to further prove the efficacy of IMRT-TBI.
